# A unifying theory for genetic epidemiological analysis of binary disease data

**DOI:** 10.1186/1297-9686-46-15

**Published:** 2014-02-19

**Authors:** Debby Lipschutz-Powell, John A Woolliams, Andrea B Doeschl-Wilson

**Affiliations:** 1 The Roslin Institute and Royal (Dick) School of Veterinary Studies, University of Edinburgh, Easter Bush, Midlothian EH25 9RG, UK

## Abstract

**Background:**

Genetic selection for host resistance offers a desirable complement to chemical treatment to control infectious disease in livestock. Quantitative genetics disease data frequently originate from field studies and are often binary. However, current methods to analyse binary disease data fail to take infection dynamics into account. Moreover, genetic analyses tend to focus on host susceptibility, ignoring potential variation in infectiousness, i.e. the ability of a host to transmit the infection. This stands in contrast to epidemiological studies, which reveal that variation in infectiousness plays an important role in the progression and severity of epidemics. In this study, we aim at filling this gap by deriving an expression for the probability of becoming infected that incorporates infection dynamics and is an explicit function of both host susceptibility and infectiousness. We then validate this expression according to epidemiological theory and by simulating epidemiological scenarios, and explore implications of integrating this expression into genetic analyses.

**Results:**

Our simulations show that the derived expression is valid for a range of stochastic genetic-epidemiological scenarios. In the particular case of variation in susceptibility only, the expression can be incorporated into conventional quantitative genetic analyses using a complementary log-log link function (rather than probit or logit). Similarly, if there is moderate variation in both susceptibility and infectiousness, it is possible to use a logarithmic link function, combined with an indirect genetic effects model. However, in the presence of highly infectious individuals, i.e. super-spreaders, the use of any model that is linear in susceptibility and infectiousness causes biased estimates. Thus, in order to identify super-spreaders, novel analytical methods using our derived expression are required.

**Conclusions:**

We have derived a genetic-epidemiological function for quantitative genetic analyses of binary infectious disease data, which, unlike current approaches, takes infection dynamics into account and allows for variation in host susceptibility and infectiousness.

## Background

Infectious diseases constitute the number one threat to livestock production, with potential devastating implications for food security and human health. With the rapid accumulation of data on the genetic regulation of host responses to infectious pathogens, the drive towards strategies that control genetic disease is gaining momentum. Genetic approaches to combat infectious disease tend to focus on improving host resistance, i.e. the ability of a host to block pathogen entry or to counteract pathogen replication within the host. However, despite enormous breakthroughs in genomics, estimating genetic parameters for disease resistance has proven considerably more challenging than analysis of production traits, and this has hampered the incorporation of disease traits into breeding programmes. These challenges partly arise because disease resistance is not a trait that is directly measurable but relies on observable proxies. Due to the requirement of large sample sizes for quantitative genetic analyses, such proxies are often obtained from field data, which are typically binary, indicating whether an individual has become infected or not [1].

Current quantitative genetic methods analyse binary infectious disease data essentially by contrasting the set of individuals diagnosed as infected to those diagnosed as non-infected, assuming that the observed phenotypic differences represent differences in host resistance to the pathogens under consideration [2]. However, the corresponding statistical models, such as threshold or logit models, entail several intrinsic assumptions that are unrealistic in the case of infectious disease: First, the observations (e.g. diseased/not diseased) are assumed to be accurate but in reality, the diagnostic tools that are used in the field rarely have complete sensitivity or specificity, i.e. there is a considerable chance for misclassification of individuals as healthy or diseased. Second, it is assumed that exposure to infectious pathogens of individuals that share the same environment is (a) equal between individuals, (b) constant over time and (c) purely environmental. However, in large groups with a non-uniform contact structure, there may be substantial heterogeneity in exposure at any given time. Thus, an individual classed as healthy may have indeed greater resistance, or could simply be misdiagnosed, or may not yet have come in contact with the infectious agents. Furthermore, for infectious diseases transmitted by direct contact, the disease status of an individual is not just the expression of its own resistance in a constant infectious environment. Instead infections result from dynamic interactions between susceptible and infected individuals, and genetic variation may be inherent to all such interactions. As the number of infected individuals in a population changes throughout the time course of a disease outbreak, exposure will change as well. Lastly, exposure depends on how infectious the infected individuals are, which may differ between individuals, e.g. due to different shedding patterns of infectious material or different durations of shedding. Thus, not only host resistance but also host infectiousness, i.e. the ability of a host to transmit an infection, may display substantial host genetic variation.

All of the above characteristics that are inherent to natural disease outbreaks are likely to affect estimates of genetic parameters for disease traits. Indeed, we have previously demonstrated that conventional quantitative genetics models fail to capture host genetic variation in infectiousness, if present [3,4]. Furthermore, theoretical work has established that imperfect diagnostics and incomplete or variable exposure produce a downward bias in estimates of heritability and of SNP (single nucleotide polymorphism) effects, and affect inferences about modes of inheritance of SNP effects for disease resistance [1,5]. This theory is empirically supported by comparing results from recent field and challenge experiments that aimed at estimating genetic parameters and at identifying genetic markers for the resistance of pigs to the Porcine Reproductive and Respiratory Syndrome Virus (PRRSV) [6,7]. Both these studies included approximately 1200 animals, but whereas infection resulted from natural transmission dynamics in the field studies [7], the challenge experiment infected all animals with the same dose of a particular PRRSV strain [6], thus excluding the various sources of heterogeneity in exposure outlined above. In accordance with theory, heritability estimates for viraemia were considerably lower based on field data than from challenge data (0.096 vs. 0.31) and the challenge study found a major QTL for disease resistance that had not been identified in the field data. Thus, both theory and experimental evidence imply that, in order to use data from natural disease outbreaks to determine the host genetic influence underlying infectious disease, current quantitative genetics methodology must be modified to take transmission dynamics into account. In quantitative genetic analyses, it is customary to assume that binary data is the realisation of a probability. Thus an important step is to identify the probability function that links the epidemiological parameters of interest, such as susceptibility and infectiousness, to the probability of becoming infected.

Therefore, the aim of this study was to derive an analytical expression for the probability of an individual to become infected within a given time period. We demonstrate how this can be achieved by integrating fundamental principles of epidemiology into the quantitative genetics framework. We then validate this analytical expression by comparing it with established theory in the case of homogeneous populations and by using simulated disease data generated for a range of epidemiological scenarios in genetically heterogeneous populations. Finally, we examine the implications for implementing this probability function into quantitative genetic analyses.

## Methods

### Epidemiological principles and approaches

The study of infectious diseases typically falls within the realm of epidemiology. A key measure in epidemiology is the basic reproductive ratio *R*_*0*_, defined as the expected number of secondary infections that one infectious individual causes in an otherwise susceptible population [8]. Efforts for epidemiological control of infections are targeted to reduce *R*_*0*_, ideally to a value below one, because if *R*_*0*_ is less than one, infection is unlikely to spread and expected to die out. The higher *R*_*0*_ is, the greater are the risk and severity of epidemics [8]. This key definition points to two important host characteristics that control the spread of infection: first, the susceptibility of non-infected individuals, i.e. the propensity of becoming infected upon contact with an infectious individual or substance, and second, the infectiousness of the infected individuals, i.e. the ability of an infected individual to transmit the infection. As stipulated by Lloyd-Smith et al. [9], for diseases transmitted by direct contact, infectiousness (or, using their terminology, individual reproductive number with population mean *R*_*0*_) can be regarded as the product of three factors: *c*, the rate at which an infectious individual comes into contact with others in the population; *f*, the probability that the disease is transmitted to a susceptible individual, given contact; and *D*, the duration of the infectious period. All three components may harbour exploitable genetic variation.

Epidemiologists rely heavily on mathematical models of transmission dynamics to predict the outcome of control strategies. For instance, using a conventional compartmental *SIR* model that describes the transition of individuals between the Susceptible (*S*), Infected (*I*) and Recovered or Removed (*R*) compartment, the change in disease prevalence is described by $\frac{\mathrm{dI}}{\mathrm{dt}}=\mathrm{\beta S}\left(t\right)I\left(t\right)-\mathrm{\gamma I}\left(t\right)$ with parameters *β* (transmission coefficient) and *γ* (recovery rate) [10]. This differential equation represents infection as a dynamic process that arises from the interaction between susceptible and infected individuals (through the use of a multiplicative term in *S* and *I*). The transmission coefficient *β* is the product of the contact rate and the probability that the contact between an infectious and a susceptible individual results in a successful transmission [10], and thus, depends on the susceptibility of the susceptible individual and the infectiousness of the infectious individual. Furthermore, for *SIR* models with constant population size, the probability *P(t)* of an initially susceptible individual to become infected within a time period *t* is given by

(1) $$P\left(t\right)=1-e^{-\Lambda \left(t\right)}$$

Where *Λ*(t) = R_0_ * R(t)/S_0_  denotes the force of infection, i.e. the rate at which susceptible individuals become infected, and *R(t)* and *S*_*0*_ are the number of recovered individuals at time t and the initial number of susceptible individuals, respectively [10].

Although epidemiologists acknowledge that there may be variation between individuals in both susceptibility and infectivity e.g. [11], classical epidemiology assumes homogeneity between individuals or within subgroups of individuals and therefore excludes the concept of host genetics. However, this gap has been shown to have a profound impact on the prediction of disease risk and prevalence, e.g. [12-14]. In particular, recent field studies have elucidated the important role of super-spreaders, the small proportion of highly infectious individuals responsible for the majority of transmission events, on the occurrence and severity of disease outbreaks across a range of diseases [15-18]. Note that super-spreaders confer host heterogeneity in infectiousness, not in resistance. Therefore, understanding and controlling heterogeneity in infectiousness, i.e. not only resistance, is now recognized as an important measure to control disease [16]. However, to date, the genetic contribution of the host to this variation in infectiousness is unknown since genetic analyses tend to focus on disease resistance and, as demonstrated in [3] and [4], fail to fully capture host genetic variation in infectiousness, if present, from binary disease data.

### Derivation of a genetic-epidemiological probability function

Binary disease phenotypes can be considered as the realization of a probability of having the observed disease phenotype. In this section, we will extend the epidemiological equation (1) for the (cumulative) probability of an individual to become infected by a time *t* for a heterogeneous host population with variation in both host susceptibility and infectiousness. For this purpose, we define *f*_*k*_ as the probability of an infectious individual *k* to infect a susceptible individual with unit susceptibility following contact, and *g*_*j*_ as the susceptibility of an individual *j* following contact with an infectious individual of unit infectivity. Furthermore, we define the indicator *X*_*f,k*_*(t)* to be equal to 1 if *k* is infectious at time *t* and to 0 otherwise. Then, the probability of a susceptible individual *j* of becoming infected following contact with individual *k* at time *t* is the product *g*_*j*_*X*_*f,k*_*(t)f*_*k*_. Let *c*_*jk*_ be the expected number of contacts in a unit time interval between individuals *j* and *k*. Thus, following the same approach as in [10], for a susceptible individual not to become infected in a unit time interval, none of the contacts must result in infection. In other words, the probability of a susceptible individual *j* to avoid getting infected in a unit time interval is equal to

(2) $$\prod_{k=1,k\neq j}^{n}{\left(1-g_{j}X_{f,k}\left(t\right)f_{k}\right)}^{c_{\mathrm{jk}}}.$$

The probability *P*_j_^*^(δt) of a susceptible individual *j* to become infected during a sufficiently short time interval *[t, t + δt]* during which the infection status of infectious individuals does not change is therefore,

(3) $$P_{j}^{*}\left(\mathrm{\delta t}\right)=1-{\left(\prod_{k=1,k\neq j}^{n}{\left(1-g_{j}X_{f,k}\left(t\right)f_{k}\right)}^{c_{\mathrm{jk}}}\right)}^{\mathrm{\delta t}}.$$

Let *P*_*j*_*(t)* be the probability of individual *j*, which was susceptible at time zero, to have become infected by time *t*. Then for a small time-step *δt,*

(4) $$P_{j}\left(t+\mathrm{\delta t}\right)=P_{j}^{*}\left(\mathrm{\delta t}\right)\left(1-P_{j}\left(t\right)\right)+P_{j}\left(t\right).$$

Note, that this equation may encompass single and repeated infections (e.g. infected, recovered and re-infected) within the time interval from 0 to *t*. Rearranging the above equation, dividing by *δt* and taking the limit *δt → 0* leads to

(5) $$\frac{dP_{j}\left(t\right)}{\mathrm{dt}}=\lim_{\mathrm{\delta t}\to 0}\frac{P_{j}^{*}\left(\mathrm{\delta t}\right)}{\mathrm{\delta t}}\left(1-P_{j}\left(t\right)\right).$$

Note that the expression for $P_{j}^{*}\left(\mathrm{\delta t}\right)$ above can be written as

(6) $$\begin{array}{c} P_{j}^{*}\left(\mathrm{\delta t}\right)=\\ 1-\exp\left(\mathrm{\delta t}\sum_{k=1,k\neq j}^{n}c_{\mathrm{jk}}\ln\left(1-g_{j}X_{f,k}\left(t\right)f_{k}\right)\right). \end{array}\;$$

Using the power series expansion of the exponential function, and dividing by δt and taking the limit *δt → 0,* leads to

(7) $$\begin{array}{ll} \lim_{\mathrm{\delta t}\to 0\;}\frac{P_{j}^{*}\left(\mathrm{\delta t}\right)}{\mathrm{\delta t}} & =-\overset{n}{\underset{k=1,k\neq j}{\sum }}c_{\mathrm{jk}}\ln\left(1-g_{j}\;X_{f,k}\left(t\right)f_{k}\right)\\ & \approx g_{j}\sum_{k=1,k\neq j}^{n}c_{\mathrm{jk}}\;\left(X_{f,k}\;\left(t\right)f_{k}\right), \end{array}$$

using the approximation ln(1 − x) ≈ − x  for small x. Substituting this last expression into the differential equation (5) yields

(8) $$\frac{dP_{j}\left(t\right)}{\mathrm{dt}}=g_{j}\;\sum_{k=1,k\neq j}^{n}c_{\mathrm{jk}}X_{f,k}\left(t\right)f_{k}\;\left(1-P_{j}\left(t\right)\right).$$

Now, define

(9) $$\Lambda_{j}\left(t\right):=\int_{0}^{t}\left(\;g_{j}\sum_{k=1,k\neq j}^{n}c_{\mathrm{jk}}X_{f,k}\left(u\;\right)f_{k}\right)\mathrm{du}.$$

so that

(10) $$\frac{dP_{j}\left(t\right)}{\mathrm{dt}}=\frac{d\Lambda_{j}\left(t\right)}{\mathrm{dt}}\;\left(1-P_{j}\left(t\right)\right).$$

Multiplying both sides of (10) so that by $e^{\Lambda_{j}\left(t\right)}$ and collecting all terms to the left hand side leads to

(11) $$\frac{d}{\mathrm{dt}}\left(\;e^{\Lambda_{j}\left(t\right)}P_{j}\left(t\right)-e^{\Lambda_{j}\left(t\right)}\right)=0,$$

or

(12) $$e^{\Lambda_{j}\left(t\right)}\;\left(P_{j}\left(t\right)-1\right)=\mathrm{constant}.$$

Hence, the solution of the differential equation (10) is

(13) $$P_{j}\left(t\right)=1+\left(P_{j}\left(0\right)-1\right)e^{-\Lambda_{j}\left(t\right)}.$$

The probability *P*_*j*_(0) can be estimated as the prevalence at the beginning of an observation period. For simplicity, however, from now on we will assume that *P*_*j*_(0) = 0 and hence,

(14) $$P_{j}\left(t\right)=1-e^{-\Lambda \left(t\right)}.$$

Note that the quantity *Λ*_*j*_(*t*) defined above can be written as

(15) $$\Lambda_{j}\left(t\right)=g_{j}\sum_{k=1,k\neq j}^{n}c_{\mathrm{jk}}f_{k}D_{k}\left(t\right).$$

where *D*_*k*_(*t*) denotes the duration of time within the interval [0,*t*] during which individual *k* is infectious. Thus, if *k* has not become infected by time *t*, *D*_*k*_(*t*) = 0, otherwise

$$D_{k}\left(t\right)=\sum_{i=1}^{m}\left(\;\min\left(t_{E_{i}},t\right)-t_{S_{i}}\right),$$

where *m* denotes the number of times that individual *k* got infected during [0,t] and $t_{S_{i}}$ and $t_{E_{i}}$ denote the start and end of the corresponding infectious periods, respectively.

### Function validation

Two forms of validation of the above derived probability function given by equation (14) with Λ_j_(t) defined in (15) were carried out. First, we assessed whether in the extreme case of zero heterogeneity in susceptibility and infectiousness, the derived function is consistent with existing epidemiological theory. Second, the function was validated with binary disease data (infected or not infected) generated by simulated stochastic epidemics in closed genetically heterogeneous populations of constant size, as described in detail in [3,4]. Two methods were chosen to illustrate this second validation: (i) a direct comparison of the probability of infection predicted by the derived analytical expressions (14) and (15) with the proportion of individuals that became infected in the simulations, and (ii) Receiver Operating Characteristic (ROC) curves. A ROC curve is a widely used graphical representation of the ability of a predictor to discriminate between cases and controls by plotting the True Positive Rate (TPR = sensitivity) against the False Positive Rate (FPR = 1-specificity) [19]. Here, the ROC curves plot the proportion of infected individuals that have an estimated probability of infection greater than a given threshold (True Positives) against the proportion of non-infected individuals that have an estimated probability of infection greater than this same threshold (False Positives). Thus, the Area Under this Curve (AUC) describes the probability of correctly ranking any infected/non-infected pair of individuals using the derived probability function. Thus, if the analytical prediction is entirely unrelated to the probability of becoming infected in the simulations, then individuals would be classified at random and the AUC would be equal to 0.5. However, if our function accurately describes the probability of becoming infected in the simulations, then the AUC would be close but not equal to 1, due to the stochastic nature of the simulations.

The stochastic epidemiological model used for validation simulates disease progression in isolated groups of *n* individuals and provides the disease status of individuals (infected/not infected) over time as output. The epidemic was simulated as a Poisson process, starting with one randomly chosen infected individual per group. The times at which subsequent infection and recovery events occurred and which individuals were affected were determined by the pairwise transmission parameters *β*_*jk*_(*t*) and by the recovery rates *γ*_*j*_(*t*), respectively, as outlined below. It was assumed that infected individuals became immediately infectious and remained infectious until they recovered. No transmission was assumed between groups.

Individual variation in host susceptibility and infectiousness was first incorporated into the model by assigning for each individual *j* its own level of susceptibility *g*_*j*_ and infectivity *f*_*j.*_ The dynamic, pairwise transmission parameter *β*_*jk*_*(t)* was then calculated as:

(16) $$\beta_{\mathrm{jk}}\left(t\right)=-c_{\mathrm{jk}}\ln\left(1-X_{g,j}\left(t\right)g_{j}X_{f,k}\left(t\right)f_{k}\right),$$

as derived in [3]. Thus, in line with standard epidemiological theory *β*_*jk*_(*t*) encapsulates the contact rate and the transmission probability. To reflect whether susceptibility and infectivity are expressed at time *t*, the individual constants *g*_*j*_ and *f*_*k*_ are scaled by *X*_*g*,*j*_(*t*) and *X*_*f*,*k*_ (*t*), respectively, which are equal to 1 if *j* is susceptible at time *t* and if *k* is infectious at time *t*, respectively, and 0 otherwise. Similarly, individual recovery rates were assumed to be equal to *γ*_*j*_(*t*) = *X*_*f*,*j*_(*t*)*γ*_*j*_, with γ_j_ and *X*_*f*,*j*_ (*t*) as defined above.

It was initially assumed that host susceptibility and infectivity were the only sources of individual variation. Thus, parameter γ_j_ was set equal to 0.1 for all individuals. For simplicity, it was further assumed that the expected number of contacts per unit time interval between two individuals in the same group was homogeneous and, without loss of generality, was set equal to *c*_*jk*_ = 1. This homogeneity assumption is likely to be satisfied in intensive farming conditions. The values of *β*_*jk*_(t) and *γ*_*j*_(t) were calculated at each event time, starting from time zero. Based on these, Gillespie’s direct algorithm was used to determine the next event (infection or recovery), the time of the event and the affected individuals, as outlined in [3]. The simulation was run until the time *t* at which approximately 50% of individuals had become infected.

In order to demonstrate that the derived probability function given by equations (14) and (15) is valid for a range of epidemiological models, binary disease data were also generated by simulating an epidemic using a stochastic SIR model with additional variation in recovery rate γ and a stochastic SLIRS model, following the same principles as described above. In a SLIRS model, the epidemiological compartments are: Susceptible (S), Latently infected but not infectious (L), Infectious (I), Recovered and temporarily immune (R), and Susceptible (S). The speed of transition between compartments S and L is given by *β*_*jk*_*(t)*, as described above. Similarly, all other individual transition speeds were assumed equal to a constant value for individuals in the relevant compartment and 0 otherwise. Specifically, the constants were; 0.5 for L → I, 0.1 for I → R and 0.2 for R → S. Similar to the previous simulation, it was assumed that the expected number of contacts between two individuals per time unit *c*_*jk*_ = 1 for all individuals from the same group. This simulation was run until the same value of *t* as above, which resulted in approximately 58% of individuals becoming infected.

Thus, the different epidemiological models used for simulation were (i) a SIR model with host variation in susceptibility and infectivity only; (ii) a SIR model with host variation in susceptibility, infectivity and recovery rate; and (iii) a SLIRS model with host variation in susceptibility and infectivity only.

Each model was run for a population of size N = 100 000 individuals, randomly divided into 10 000 isolated groups of size 10 chosen, which is equivalent to simulating 10 000 independent epidemics. Susceptibility and infectivity were assumed to be distributed according to a right-skewed gamma distribution Г(*a,θ*), which is representative for a variety of infectious diseases [16]. Moreover, skewed distributions allow for larger variation when the distribution is confined to positive values. For simplicity, susceptibility and infectivity were assumed to be independent. Similarly, additional individual variation in recovery rate was incorporated into the above described SIR model by sampling individual time to recovery 1/*γ*_*j*_ from a right-skewed Gamma distribution Γ(2,5). In other words, it was assumed that most individuals recover quickly, that a few individuals may take a very long time to recover, and that the mean time to recovery was ten time units. This simulation was run until the same value of *t* as above, which resulted in approximately 41% of individuals becoming infected.

Each epidemiological model provided the binary disease state (infected/not infected by time *t*) for every individual as output. Furthermore, the period of time during which each individual remained infectious (D_k_) was recorded for validation purposes. Note that the duration of the infectious period D in equation (15) captures individual variation in the transmission speeds between compartments I, I R and RS. Knowledge of the infectious period, together with the known input values of c, *g* and *f*, allowed calculation of the quantity Λ_j_(t) using equation (15) and hence the probability of becoming infected by a time *t*, based on equation (14). This was then compared with the observed proportion of individuals that became infected by time *t* in the simulations, within a given class of Λ_j_(t). The class size for Λ_j_(t) was taken as 0.02 to ensure that sufficient records were available within each class.

## Results

### Validation of the probability function

#### Concordance with epidemiological theory

We first demonstrate that for homogeneous populations, equations (14) and (15) are consistent with existing epidemiological theory and with the method of survival analysis. In a homogeneous population, i.e. when there is no variation in susceptibility (g_j_ = g for each individual j), infectivity (f_k_ = f for all k), contact rate (c_jk_ = c for all j, k) or any of the other epidemiological parameters, equation (15) becomes

(17) $$\Lambda_{j}\left(t\right)=\Lambda \left(t\right)=\;\mathrm{cgf}\;\sum_{k=1,k\neq j}^{n}D_{k}\left(t\right).$$

Also, following equation (16), in the case of homogeneity, for any pair consisting of a susceptible individual *j* and an infectious individual *k* (i.e. X_g,j_(t) = X_f,k_(t) = 1), the transmission coefficient is

(18) $$\beta =-\mathrm{cln}\left(1-\mathrm{gf}\right)\approx \mathrm{cgf},$$

for small values of *g* and *f*.

Furthermore, the sum of the infectious period of each individual in a group, within the time interval from 0 to *t,* can be written as

(19) $$\sum_{k=1,k\neq j}^{n}D_{k}\left(t\right)=\int_{0}^{t}I\left(\tau \right)\mathrm{d\tau},$$

where I(τ) denotes the number of infectious individuals at time τ. In an SIR model with constant recovery rate γ, the number of recovered individuals, R, changes over time according to *dR*/*dt* = *γI*(*t*), thus yielding the following for the above sum over infectious periods

(20) $$\sum_{k=1,k\neq j}^{n}D_{k}\left(t\right)=\frac{1}{\gamma }R\left(t\right).$$

Note that in an SIR model, the basic reproductive ratio R_0_ is

(21) $$R_{0}=\beta \frac{S_{0}}{\gamma },$$

where S_0_ is the number of susceptible individuals at the start of the epidemic [10]. Substituting equations (18) to (21) into (17), yields for Λ_j_(t) = Λ(t)

(22) $$\Lambda \left(t\right)=\frac{R_{0}\;R\left(t\right)}{S_{0}},$$

and hence for P_j_(t) = P(t) according to equation (14)

$$P\;\left(t\right)=1-\exp\left(-\frac{R_{0}\;R\left(t\right)}{S_{0}}\right).$$

Hence, the expression for the probability of becoming infected derived, as in the paragraph “Epidemiological principles and approaches” for heterogeneous populations, i.e. equation (14), is consistent with equation (1) from epidemiological literature if there is no individual variation.

The probability function (14) is also consistent with the notion of failure in survival analysis, where the failure function F(t) represents the probability of failure by time *t* and is defined as *F*(*t*) = 1 − *e*^−*Λ*(*t*)^, where Λ(t) is the cumulative hazard function [20]. In this context, failure represents becoming infected. Therefore, equation (14) can be considered a failure function with a cumulative hazard function given by equation (15).

#### Function validation with simulated disease data

**Figure 1:**
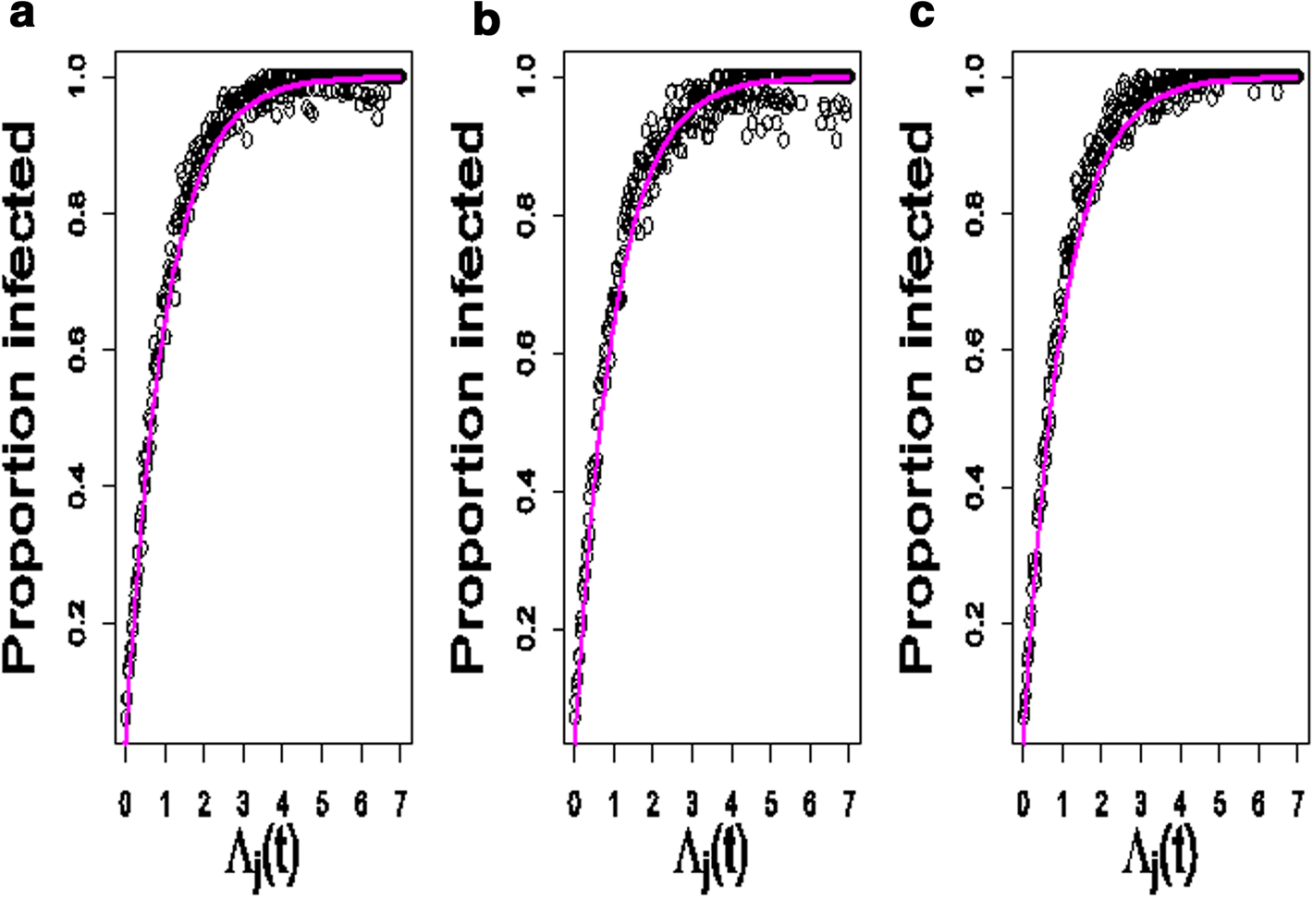
**Comparison of the probability function (equations (14) and (15)) with results from simulated disease data.** For details regarding simulation parameters see paragraph “Derivation of a genetic-epidemiological probability function“; data points: proportion of infected individuals for a given class of Λ_j_(t) using equation (15) with class size 0.02; curve: expected probability of becoming infected by time *t* following equations (14) and (15); panels: **a**. SIR model with variation in susceptibility and infectivity only, **b**. SIR model with variation in recovery rate, and **c**. SLIRS model.

Figure 1 shows the proportion of individuals that had become infected by time *t* in the epidemiological simulations, for a given time *t* and calculated values of Λ_j_(t), as well as the analytical expression for the probability of becoming infected derived in equations (14) and (15). Figures 1a,b and c indicate that the probability function provides a good fit to the probability of becoming infected. Moreover, this function provides a robust fit across a range of epidemiological scenarios, as shown in Figures 1a,b and c for, respectively, the SIR model with variation in susceptibility and infectivity, with additional variation in recovery rate, and the SLIRS model. Note that parameter values used in the simulations (see the above paragraph “Derivation of a genetic-epidemiological probability function”) are arbitrary and not expected to affect the fit.

**Figure 2:**
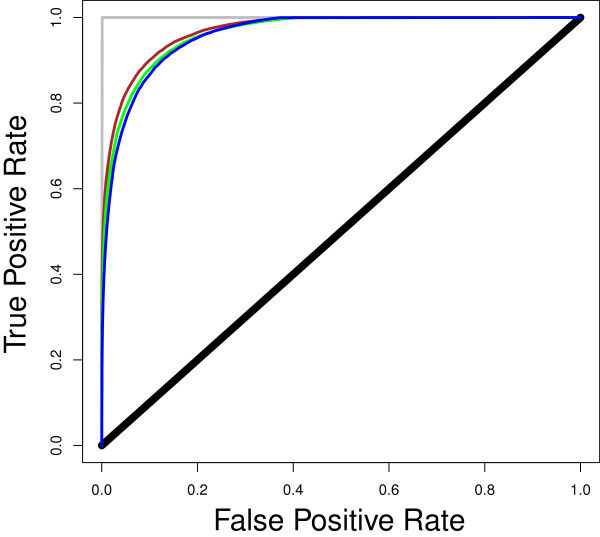
**ROC curves for predicting disease status using the probability function (equations (14) and (15)).** Curves: green = data from simulation of the SIR model with variation in susceptibility and infectivity (AUC = 0.964); blue = data from simulation of SIR model with variation in susceptibility, infectivity and recovery rate (AUC = 0.960); brown = data from simulation of SLIRS model with variation in susceptibility and infectivity (AUC = 0.970); black = random classification (AUC = 0.5); grey = perfect classification (AUC = 1).

Figure 2 shows ROC curves for predicting whether an individual has become infected or not by time *t*, with the derived probability given by equations (14) and (15) as the classification criterion. According to Figure 2, the derived probability is effective at predicting whether an individual will become infected or not by time *t*, in a manner that is consistent with an accurate probability function, i.e. with an AUC that is close to, but not equal to, 1. Moreover, the predictive ability of the derived probability function is robust across a range of epidemiological scenarios, with an AUC between 96-97% for all simulations.

**Figure 3:**
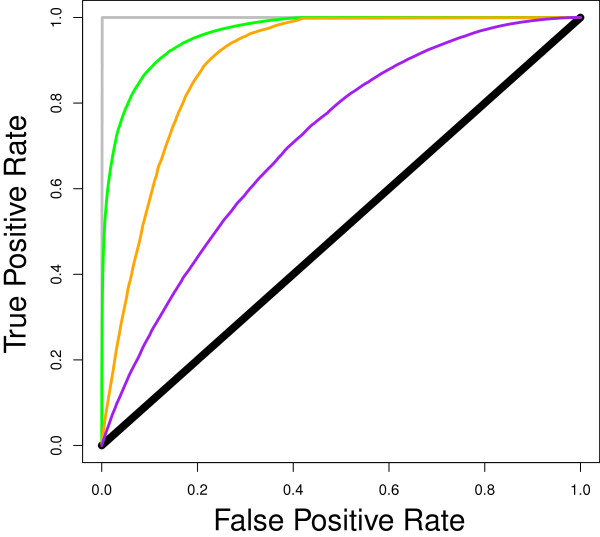
**Effect of including different sources of host variation on the prediction of individual disease status.** ROC curves calculated with data from simulation of the SIR model with variation in susceptibility and infectivity; the classification criterion used was the probability function equation (14) with Λ_jt_ including different sources of variation; curves in green = ‘**Genetic epidemiology**’ - Λ_jt_ includes all sources of variation and was estimated based on equation (15) (AUC = 0.964); orange = ‘**Epidemiology**’ - Λ_jt_ was estimated assuming no (genetic) variation in susceptibility and infectivity, as in equation (17) (AUC = 0.895); purple = ‘**Genetics**’ - Λ_jt_ was estimated assuming (genetic) variation in susceptibility and infectivity, but equal non-dynamic exposure, i.e. $D_{k}\left(t\right)=\bar{D}$ for each individual *k* (AUC = 0.710); black = random classification (AUC = 0.5); grey = perfect classification (AUC = 1).

The probability function (14), with Λ(t) defined in (15), captures different sources of host (genetic) variation, which may not be easy to estimate in practice. In particular, whereas susceptibility *g* and infectivity *f* may harbour substantial genetic variation, the duration of the infectious period *D* within a given time interval are more likely to depend upon a combination of various genetic (e.g., *g*, *f* and also in γ) and environmental (e.g., choice of time interval), or other stochastic factors. In order to determine the importance of estimating these components of Λ_j_(t) for predicting the future disease status of an individual, ROC curves were also generated with the classification criterion estimated by assuming either no (genetic) heterogeneity in *g* and *f* (i.e. calculating Λ_j_(t) according to equation (17)), or by assuming genetic heterogeneity but equal non-dynamic exposure ($D_{k}\left(t\right)=\bar{D}\;$ for each individual *k*) in the probability function. The first scenario may be considered to be in line with current epidemiological theory, as outlined in the above paragraph “Derivation of a genetic-epidemiological probability function“ (equation (17)), whereas the second scenario may be considered to be more in line with current quantitative genetics theory that ignores dynamic exposure. Note that exact values of D_k_(t) may not be available from field data and, therefore, using the further approximation from equation (20) is more in line with current epidemiological practice. However, applying this approximation results in discrete values of D_k_(t) rather than a continuous curve (results not shown). Nonetheless, the resulting discrete values are close to the curve obtained without using this approximation. Figure 3 shows a comparison of the ROC curves that correspond to these ‘epidemiological’ and ‘genetic’ assumptions, with the ROC curve that combines genetics and epidemiology in the derived expression for Λ_j_(t) outlined in equation (15). The ROC curves in Figure 3 reveal that quantifying the exposure over time explains most of the ability to predict whether an individual will become infected or not. Furthermore, predictions of an individual’s disease status are considerably improved when all sources of genetic and epidemiological variation are included in the calculations.

## Discussion

### Extension to current epidemiological and quantitative genetics theories

Using mathematical principles, a genetic – epidemiological probability function was derived that links binary disease data to the underlying epidemiological traits, host susceptibility and infectiousness. The function is an extension of the established epidemiological equation for the probability of becoming infected by a time *t* (1) from homogeneous to heterogeneous populations. Indeed, in line with epidemiological theory, the quantity Λ_j_(t) described in equation (15) may be called *the individual force of infection* of an individual *j* at time t. Defining infectiousness of individual *k* towards individual *j* until time *t* as the product ϕ_*jk*_(*t*) = *c*_*jk*_*f*_*k*_*D*_*k*_(*t*), as previously postulated by Lloyd-Smith et al. [9], simplifies the expression for Λ_j_(t) to:

(23) $$\Lambda_{j}\left(t\right)\;=g_{j}\;\sum_{k=1,k\neq j}^{n}\phi_{\mathrm{jk}}\left(t\right).$$

Thus, the force of infection for an individual *j* is the product of the individual’s susceptibility and the cumulative infectiousness of its group members towards it, which reflects that an infectious disease results from interactions between susceptible and infectious individuals. Note that under the assumption that *c*_*jk*_ = *c*_*k*_ for each individual *k*, the infectiousness *ϕ*_*jk*_(*t*) derived here corresponds to the individual reproductive number with population mean R_0_, as defined in epidemiological literature [9]. In the context of quantitative genetics, the cumulative infectiousness replaces the concept of exposure. Rather than an equal, constant and purely environmental exposure, as is typically assumed [5], the individual force of infection in equation (23) illustrates that exposure depends on the number of infectious individuals, which may change over time as their infection status changes, as well as on their contact behaviour and infectivity, where some or all of these components may be partly genetically determined. In particular, the time *D*_*k*_*(t)* during which an individual remains infected may be partly genetically determined since it encapsulates several mechanisms that are determined by the immune system, such as recovery and latency. Thus, there is potentially much to be gained by incorporating epidemiological information into genetic analyses, and vice-versa, as illustrated in Figure 3.

The concept that an individual’s phenotype is not only controlled by its own genes but also by the genes of interacting individuals is not new in quantitative genetics, and has already been successfully incorporated in the form of indirect (or associative) genetics effect (IGE) models [20-22]. We have previously applied such IGE models to estimate genetic parameters associated with host susceptibility and infectivity from simulated binary disease data [3,4], and found that IGE models can indeed capture some of the genetic variation underlying infectiousness. However, we have also found use of the current IGE framework in the context of infectious disease to have shortcomings since crucial dynamic aspects are ignored, which leads to bias in parameter estimates [4]. As outlined in more detail below, the derived genetic-epidemiological probability function offers a means to extend the current IGE model framework to infectious diseases in populations that display genetic variation in diverse epidemiological traits for which expression varies throughout the time course of infection.

### Implementation of the probability function into quantitative genetic analysis

In order to incorporate susceptibility and infectiousness into genetic selection programs, knowledge of the respective genetic (co)variances is required. Moreover, it might be desirable to use estimated breeding values of these traits for genetic selection or for genome-wide association studies. Estimation of breeding values by best linear unbiased prediction requires not only knowledge of the genetic variance [2] but also the use of mixed models, as these allow simultaneous estimation of fixed effects and random genetic effects [2]. Susceptibility and infectiousness are difficult to measure directly and, as was assumed in this paper, field disease data is often binary, indicating whether an individual became infected or not. It is customary to use a generalized linear (mixed) model (GL(M)M) to analyse binary or categorical data [23]. In such models, the observed trait is linked to an assumed linear model of the underlying continuous trait(s) via a non-linear link function. Canonical link functions that are commonly used for binary data are the probit and logit link functions [23], which assume that the probability of the trait to be equal to one, i.e. to have become infected in our case, follows a cumulative normal or a logistic distribution, respectively [23]. Despite their convenient mathematical properties, neither distribution, however, arises naturally from epidemiological theory, as demonstrated in the present study. A consequence of this is that interpretation of such analyses in terms of epidemiological parameters is problematic at best. A suitable link function for a GL(M)M transforms the observed trait into a linear expression of the parameters of interest. However, in the genetic epidemiological probability function P_j_(t) (equation (14) with Λ_j_(t) defined in equation (23)), the parameters of interest, i.e. the epidemiological traits susceptibility and infectiousness, enter in a multiplicative rather than in a linear manner. However, if there was genetic variation in susceptibility *only*, it follows from equations (14) and (23) that the probability P_j_(t) can be linked to the following linear model in susceptibility using a complementary log-log link function:

(24) $$\ln\left(\Lambda_{j}\left(t\right)\right)\;=\ln\left(g_{j}\right)+\ln\left(\sum_{k=1,k\neq j}^{n}\phi_{\mathrm{jk}}\left(t\right)\right).$$

Assuming no genetic variation in the epidemiological traits *cjk*, *fk* and *Dk* that underlie infectiousness, the second summand of equation (24) can be considered to be an error term e_j_(t). However, in contrast to using the canonical logit and probit link functions, this model captures and completely separates the individual’s susceptibility from the dynamic aspects of exposure.

However, when there is genetic variation in both susceptibility and infectiousness, it is not straightforward to link the probability P_j_(t) of becoming infected to a linear model that includes both susceptibility and infectiousness. Indeed, the complementary log-log link function (24) is no longer adequate when there is variation in infectiousness since the logarithm of a sum does not equal the sum of the logarithms. It is, however, possible to linearize the force of infection from equation (23), in both susceptibility and infectiousness, using e.g. the Taylor series expansion of $\Lambda_{j}\left(t\right)=g_{j}\sum_{k=1,k\neq j}^{n}\phi_{k}\left(t\right)\;$ near the population mean susceptibility $\bar{g}$ and the population mean infectiousness $\bar{\phi }\left(t\right)\;$ up to time *t*:

(25) $$\begin{array}{l} \begin{array}{ll} \Lambda_{j}\left(t\right) & =\left(n-1\right)\bar{g}\;\bar{\phi }\left(t\right)+\left(n-1\right)\bar{\phi }\left(t\right)\left(g_{j}-\bar{g}\right)\\ & +\bar{g}\sum_{k=1,k\neq j}^{n}\left(\phi_{\mathrm{jk}}\left(t\right)-\bar{\phi }\left(t\right)\right) \end{array}\\ \;+\left(g_{j}-\bar{g}\;\right)\sum_{k=1,k\neq j}^{n}\left(\phi_{\mathrm{jk}}\;\left(t\right)-\bar{\phi }\left(t\right)\right) \end{array}$$

Note that the Taylor series of Λ_j_(t) in equation (25) is not truncated and that it includes only one non-linear term in susceptibility and infectiousness. Following a GL(M)M framework, if the last term of equation (25) was negligible, the expression for Λ_j_(t) would be linear and thus an appropriate link between observed binary disease data (infected or not infected) and the underlying epidemiological traits, host susceptibility and infectiousness.

**Figure 4:**
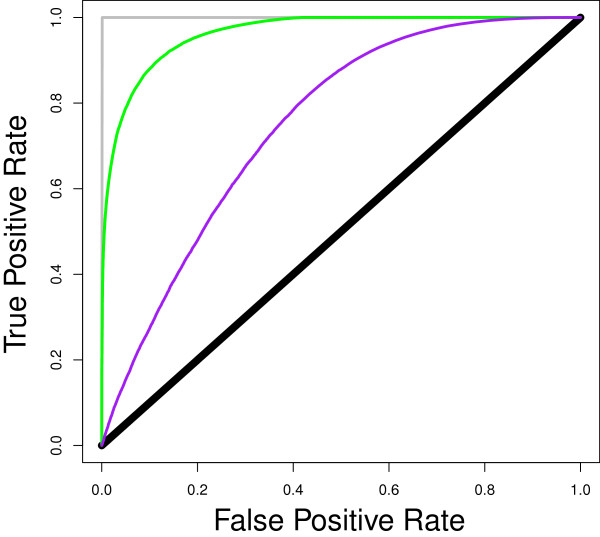
**ROC curve for predicting disease status using an IGE model.** Data from simulation of the SIR model with variation in susceptibility and infectivity; curves in green = the probability function with lambda estimated as in equation (15) used as classification criterion (AUC = 0.964); brown (overlapping with green curve) = the probability function with lambda estimated using the Taylor expansion from equation (25) used as classification criterion (AUC = 0.964); purple = an IGE model (equation (26)) used as classification criterion (AUC = 0.751); black **=** random classification; grey = perfect classification.

Note that truncating equation (25) after the linear terms in *g*_*j*_ and *ϕ*_*jk*_(*t*) corresponds to an IGE model for the individual force of infection Λ_j_(t). IGE models describe the phenotype P_j_ (here P_j_ = Λ_j_(t)) of an individual *j* as a linear combination of the individual’s direct effect P_Dj_, and the cumulate indirect (or associative) effect P_Sk_ of its group members, i.e.

(26) $$P_{j}\left(t\right)=\mu +P_{D_{j}}+\sum_{k=1,k\neq j}^{n}P_{S_{k}},$$

with an underlying genetic component for both the direct and indirect effects and with μ denoting the population mean phenotype, e.g. [20,21]. The connection between host infectiousness and indirect effects has been established previously [3] but the exact nature of this connection was unknown. Thus, comparison of the linear part of equation (25) with equation (26) offers a new interpretation of direct and indirect effects in this context and of previous results. Indeed, according to equation (25), the direct effect corresponds to the susceptibility of individual *j* (expressed as deviation from the population mean susceptibility), scaled by the cumulative average infectiousness of the group members up to time *t*, and the indirect (or associative) effect of a group member corresponds to its infectiousness (expressed as deviation from the population mean infectiousness until time *t*), scaled by the average population susceptibility. Furthermore, equation (25) may shed some light on potential causes for the previously observed bias in the genetic parameter estimates in infectivity [4]. This bias may have resulted from the inadequacy of the linear and logit models used in the previous analyses, as neither emerges from epidemiological theory and the appropriate link function was yet unknown. Furthermore, as illustrated in equation (25), the non-linear interaction between susceptibility and infectiousness may become non-negligible if there are large deviations in infectiousness *ϕ* from the population mean. This is illustrated in Figure 4, which shows the ROC curves with the classification criterion estimated with the full (AUC = 0.964) and truncated (AUC = 0.751) versions of equation (25). In other words, in the presence of super-spreaders, i.e. highly infectious individuals, the use of a GL(M)M or any other linear framework is likely to create bias. For the purpose of identifying super-spreaders, it would therefore be desirable to develop computational algorithms that do not require linear approximations of the force of infection function. Such non-linear algorithms would also be needed to disentangle the individual components of infectiousness, e.g. to separate genetic variation in the ability to transmit the infection upon exposure (i.e. variation in *f*) from genetic variation in the duration of the infectious period (i.e. variation in *D*). These sources of variation likely correspond to different immunological processes (e.g. shedding vs. recovery) and may therefore be controlled by different sets of genes. However, separating infectiousness components in genetic analyses may come with additional data requirements. For example, repeated binary measurement of an individual’s disease status over time rather than one single snapshot in time may be required to infer genetic variation in the duration of the infectious period. These measurements may be taken from on-going epidemics by using equation (13) instead of (14), with P_j_(0) equal to the prevalence of the disease in the first observation. Markov Chain Monte Carlo methods [24], with their hierarchical iterative sampling process, appear well suited to incorporate the dynamic expression of host susceptibility and infectiousness. Such methods may also lend themselves more easily to the consideration of other uncertainties that frequently affect observed disease phenotypes, such as incomplete sensitivity or specificity of diagnostic tests.

## Conclusions

We have derived a genetic epidemiological function for quantitative genetic analyses of binary infectious disease data that takes genetic variation and the dynamic expression of host infectiousness into account. The function describes the probability of an individual to become infected given its own susceptibility and the infectiousness of its group mates. When variation is limited to host susceptibility, it is possible to estimate genetic variation for this trait in a manner compatible with epidemiological dynamics using the complementary log-log link function. When there is genetic variation in both susceptibility and infectiousness, it is possible to use the logarithmic link function with a linear IGE model but this is likely to generate prediction bias if there is a large variation in infectiousness. Future work will concentrate on developing computational algorithms that can incorporate the genetic epidemiological function without linear approximations, in order to identify potential genetic super-spreaders. These algorithms would enable us to uncover the genetics underlying epidemics and thus shape the epidemics of tomorrow.

## Competing interests

The authors declare that they have no competing interests.

## Authors’ contributions

DLP carried out and extended the initial outline of the derivations, wrote the simulation program, carried out the validation, and drafted the manuscripts. JAW conceived the study with an initial outline of derivations, and helped to draft the manuscript. ABDW led the supervision, steered the development and interpretation of the study, and carried out major revisions to the manuscript in the editing process. All authors read and approved the final manuscript.
